# Evidence-based design of vaccine hesitancy information resources for health care providers

**DOI:** 10.5195/jmla.2026.2135

**Published:** 2026-10-01

**Authors:** Margaret Ansell, Hannah F. Norton, Nina C. Stoyan-Rosenzweig, Jane Morgan-Daniel, Mary E. Edwards, Lauren Adkins

**Affiliations:** 1 meansell@ufl.edu, Associate Chair, Health Science Center Libraries, University of Florida, Gainesville, FL; 2 nortonh@ufl.edu, Chair and Fackler Director, Health Science Center Libraries, University of Florida, Gainesville, FL; 3 nstoyan@ufl.edu, Archivist and Senior Associate in Libraries, Health Science Center Libraries, University of Florida, Gainesville, FL; 4 morgandanie.jane@ufl.edu, Associate University Librarian, Health Science Center Libraries, University of Florida, Gainesville, FL; 5 meedwards@ufl.edu, University Librarian, Health Science Center Libraries, University of Florida, Gainesville, FL; 6 lauren.adkins@ufl.edu, Associate University Librarian, Health Science Center Libraries, University of Florida, Gainesville, FL

**Keywords:** Vaccine hesitancy, clinical information resources, evidence-based librarianship, qualitative data analysis

## Abstract

**Background:**

The University of Florida Health Science Center Libraries (HSCL) sought to address the increasing challenge of vaccine hesitancy during the COVID-19 pandemic by developing evidence-based information resources for health care providers. To do this, HSCL librarians assessed the vaccine hesitancy information needs of local health care providers to design a resource that could be used to support conversations with vaccine hesitant patients at the point of care.

**Case Presentation:**

A team of six librarians, supported by student research assistants, conducted interviews with 20 health care providers in north central Florida. The interviews, conducted via Zoom, focused on providers' experiences with vaccine-hesitant patients. Content analysis of the transcribed interviews identified key themes, including trust, individualized conversations, and the impact of misinformation. The team developed a resource guide based on these findings, featuring curated information to support providers in vaccine discussions.

**Conclusions:**

The results highlighted the importance of trust in patient-provider relationships and the challenges posed by misinformation. Providers expressed a need for diverse, accessible resources to aid in conversations about vaccines. The resulting resource guide, disseminated through local health networks, aims to enhance providers' ability to address vaccine hesitancy effectively. This project underscores the critical role of librarians in public health initiatives and the value of collaborative efforts to combat vaccine hesitancy.

## BACKGROUND

The University of Florida Health Science Center Libraries (HSCL) are committed to engaging with local communities and promoting access to trustworthy health information. However, the libraries’ position serving academic and clinical populations means that the librarians have little direct contact with the public. Instead, through HSCL’s community engagement strategy, library personnel have focused on developing strong partnerships with public-facing local health and social service organizations who are trusted by community members. Libraries, as information providers, are well placed promote access to scientific research and writings on vaccination and on historic concerns about vaccination processes. [1] Prompted by the COVID-19 pandemic, in 2021–2022 a team of HSCL librarians was awarded an internal grant to assess the vaccine hesitancy information needs of health care providers [2].

The University of Florida is in Alachua County located in north central Florida. North Central Florida’s population is often categorized into three groups: 1) the small town and university area of Gainesville, which is demographically younger, more diverse, and more highly educated; 2) the retirement area south of Gainesville that has a higher proportion of elderly residents; 3) and the rural counties to the north, east, and west of Gainesville. Like many other rural areas, access to health care is a challenge, exacerbated by the percentage of families living at or under the federal poverty level and the number of immigrants, particularly rural migrant laborers [3]. These populations also contain groups who are skeptical of health care providers and may be vaccine hesitant [4].

The goal of this project was to develop a resource to help improve the quality of conversations that health care providers have with their patients. We aimed to design the resource by obtaining a better understanding of the reasons for vaccine hesitancy and the sharing of information resources addressing these reasons. This was done through an information needs assessment in which local health providers and advocates were interviewed and those interview transcriptions analyzed. The project team sought to interview a variety of individuals who regularly have conversations with patients about vaccines, with a goal of twenty interviews that represented at least three different provider or advocate types.

## CASE PRESENTATION

### Methods

The grant funding allowed for the hiring of student research assistants to collaborate with the librarian project team members. The six librarians focused on developing the project plan and methodology, using professional connections to recruit and make initial contact with potential study participants, conducting interviews, and leading data coding and analysis. The project team targeted providers who were known to have regular conversations about vaccines with their patients, such as primary care providers, but were broad in their definition of ‘provider’ and reached out to contacts in various medical specialties at UF Health and health advocates in the surrounding community. Use of an onboarding checklist and training session ensured that the research assistants understood the context of the project, as well as the process of conducting qualitative research. While the concept of validity in qualitative research has been considered controversial, Maxwell [5] discusses strategies to prevent validity threats, several of which were utilized in this project, including the use of rich data (meaning detailed and in-depth data that can be used to deeply understand the meaning behind experiences or phenomena) and applying numbers to assess the amount of evidence in the data. The research assistants participated in drafting the interview questions, facilitated the logistics of scheduling interviews with study participants, recorded and transcribed the interviews, and participated in the coding and qualitative analysis of the transcripts. The project plan was submitted to the University of Florida’s institutional review board and was categorized as exempt (IRB #202101941).

#### Data Collection

To make interviews as accessible as possible to busy health care providers, and to take advantage of computer-generated transcription features, the project team conducted interviews over Zoom. The project lead developed a protocol document (see Appendix) that was used to guide interview setup and procedure, ensuring consistency across all interviews. It also helped the team remember important details like ensuring that the online consent form was completed, recording the interview, and asking for contact information to deliver the participant incentives. As a semi-structured interview, the team developed a list of core questions to ask about provider experiences with vaccine conversations, particularly ones in which patients are vaccine hesitant:

1. Do you talk to your patients about getting vaccines for: 1) themselves, 2) their children, or 3) other loved ones (parents, etc.)? How do you approach these conversations?
2. Do you encounter people who hold beliefs that may make them hesitant to get vaccines? What strategies do you use to discuss their beliefs with them?
3. Where do you think patients receive the most influential information about vaccines from?
4. What factors have you seen/ do you think influence a person’s decision on whether to get a vaccine or not?
5. How do you decide which resources are most suitable to recommend for particular patients, in relation to vaccines?
6. In your opinion, what are the best ways to present vaccine information to patients?
7. What resources or tools would make these conversations easier for you?

The protocol also included a list of supplementary questions that interviewers could use to elicit additional information, depending on how detailed and informative the initial responses were. Interviewers had the flexibility to pursue additional areas of inquiry based on participant responses.

Librarians identified potential participants through existing contacts who were likely to work with vaccine hesitant patients. This included people from the team's liaison groups and past projects who were either institutionally affiliated or community-based health care providers. Health care provider was defined broadly as any professional involved in the health care process who has conversations with patients about vaccines on a consistent basis.

#### Data Analysis

Zoom transcripts were corrected to capture verbatim conversations. The use of full transcripts and “rich data” is one of several strategies for dealing with threats to validity in qualitative research. [5] To best fit the type of data and project goals, the research team elected to use a collaborative approach to qualitative data analysis. [6] The process included multiple rounds of coding, review, and triangulation to ensure consensus amongst the coders and consistency of code application (Figure 1). The full coding protocol is available at: https://osf.io/f875j/files/t8a6k.

**Figure 1:**
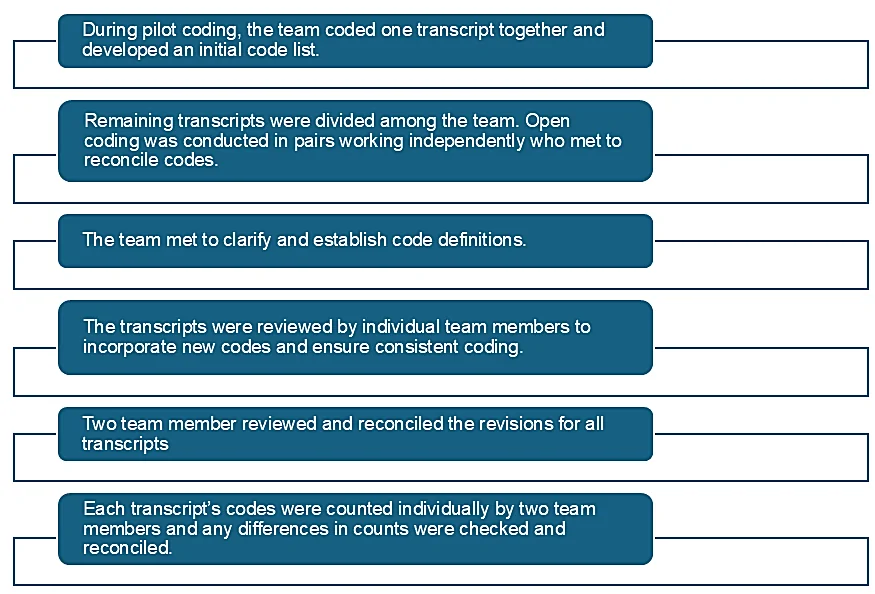
Approach to Qualitative Data Analysis

To obtain additional direction related to the design of an information resource, the team performed a secondary analysis of the interview questions targeting information resources. As a first step, one team member created a new document collating excerpts from each interview transcript that specifically mentioned particular information resources. The rest of this process was a streamlined version of the primary analysis with some high-level themes and recommendations being derived from the more focused dataset.

### Findings

#### Participant Demographics

The team interviewed a total of 20 individuals, including clinicians, administrators, community health workers, and community health advocates. Many of the physician participants who responded are in specialties in which recommendations about vaccines feature regularly in clinical settings, such as pediatrics and family medicine. Several of the physician participants also spoke about their experiences in the context of vaccine-related projects or outreach work that they had begun during the pandemic.

#### Data Analysis

Using iterative content analysis for the primary analysis, the team created 86 unique inductive codes that were applied to interview transcripts. Those codes were tagged to transcripts a total of 1,725 times, with an average of 86 code instances per transcript. The same code could be tagged multiple times within each transcript.

Several codes were grouped into larger categories. These broader categories include the following:

- **Demographics**: age, education, socioeconomic status, race/ethnicity, religion, rural/urban
- **Information Content**: patient experiences/testimonials, data
- **Information Format**: audio, infographic, language, messaging, phone, print, video
- **Information Sources**: celebrities, experts, government websites, news media, research data, scholarly literature, social groups, social media
- **Reasons for Vaccine Hesitancy**: access/logistics barriers, conspiracies, fear, ID requirement, medical intervention hesitance, low individual risk, survived COVID, patient family/friends’ experiences, political ideology, safety concerns, religious beliefs, skeptical of effectiveness, social groups, side effects
- **Reasons for Vaccination**: multiple/complex/overlapping rationale, high individual risk, patient family/friend experiences, protecting friends/family, religious beliefs, vaccine mandate

The most commonly applied codes across transcripts were: “providers reducing barriers: reframing the conversation” (93 instances), “individualized conversations” (73), and “trust” (62), and “patient provider relationship” (57). All codes used at least 20 times are shown in Figure 2.

**Figure 2:**
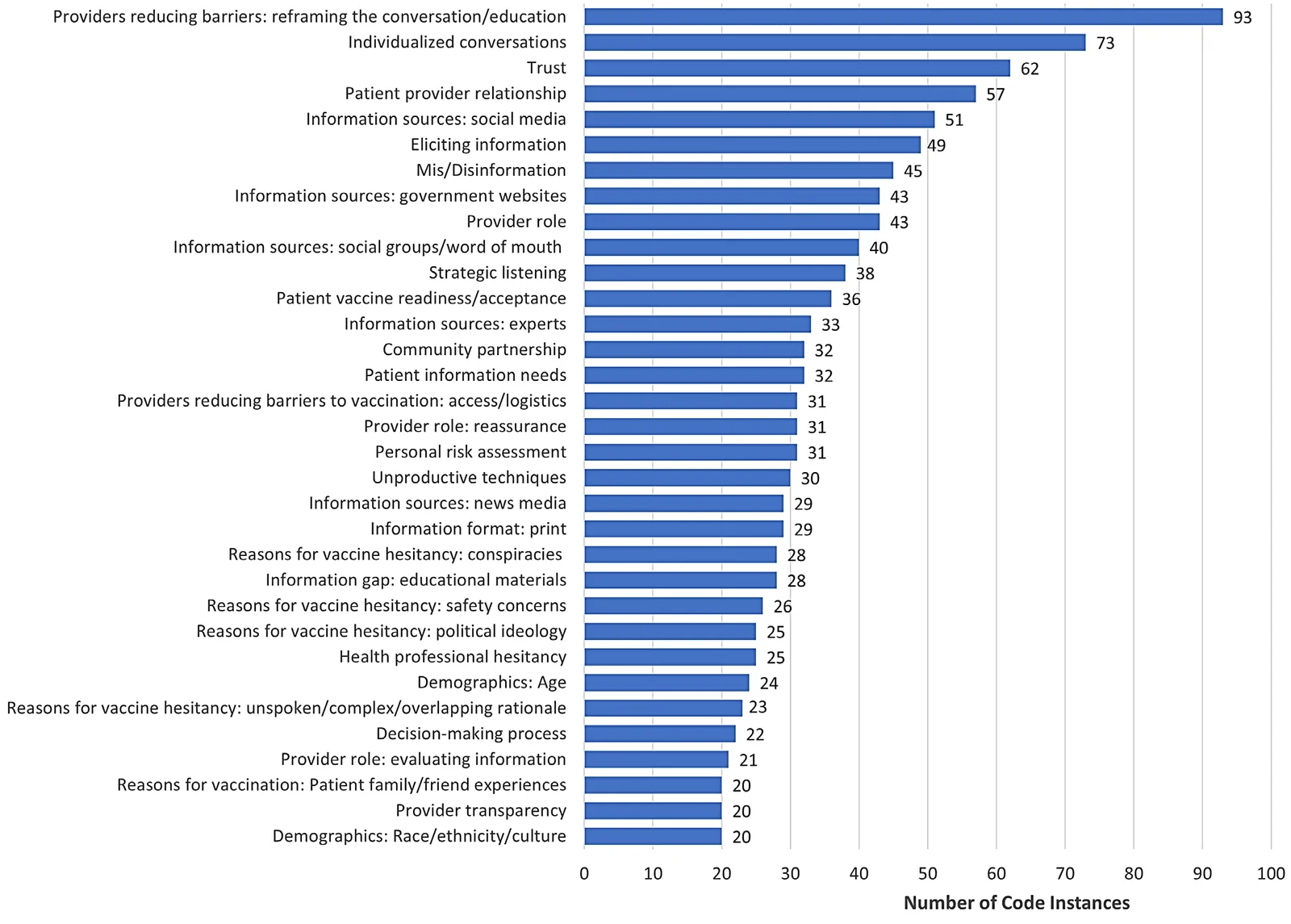
Number of Uses of Codes Across All Transcripts. Only codes with 20 or more uses are included.

The code “individualized conversations” was defined as “interviewees customizing the vaccination conversations based on their own individual experiences and beliefs”. For example, one interviewee mentioned that sharing personal experiences one-on-one to establish a connection is essential:

“I think for really difficult, intractable issues like this, then the effort of going and trying to talk to people individually and trying to share those experiences, one on one. You know not talking at people and not trying to get them to come to one place, but really meeting them where they are, is super important - [and] worth the effort that it takes.” - Interviewee #17, Community Health Worker

Another interviewee discussed the importance of having genuine conversations with people instead of having a canned response that could across as inauthentic:

“I approach all my vaccine conversations based around what the individual person specifically has questions about or is hesitant about. So, I don’t give some canned spiel about how vaccines are safe and effective, I ask people ‘What questions do you have? What is [the] cause in your hesitation?’ and I try to invite them to share anything and everything and I say that there is no concern that is too silly, there is no question that is at all a bad question.” - Interviewee #20, Medical Director of a local clinic

Interviewees’ responses pertaining to this code suggest that tailoring “individualized conversations” was considered an essential aspect of successful provider-patient communications related to vaccine hesitancy, particularly in terms of establishing personal connections and addressing specific concerns and questions.

The code “providers reducing barriers: reframing the conversation” was defined as “providing information in a way that helps patients better understand the benefits and risk of vaccination”. Examples included asking patients why they were not completely vaccine-averse and reminding them of the potential impacts of vaccination on family members.

“I’ll ask if they're interested in talking about the COVID vaccine like 'Where are you on a scale one to 10 for readiness to get the COVID vaccine?' If they’re a 2, then, sometimes you'd like to get some positives out of them you'll say 'Okay, well, why aren't you a 1?'” - Interviewee #2, Pediatric Endocrinologist

“'You're pretty healthy, but your elderly mom who you live with, who has contact with you daily who's not going out and being exposed to anybody else but you're going out and being exposed to everybody else, if you got it [the vaccine], you could really help her.'” - Interviewee #6, Community Medicine Doctor in a rural area

The code “trust” was defined as “indicates a specific mention of either the presence or lack of [trust], often as an element of a patient/provider relationship”. Discussed further below in the context of information sources, trust was considered a key element of successful vaccine conversations.

“For my specific patients…who have long standing trusting relationships with me, a lot of these folks ending up getting vaccinated despite how concerned they were about a lot of things because of the level of trust. Like it was the trust; the trust was the most important thing. It wasn’t even how well I made the argument or the logic behind it. It was really, at the end of the day, it was they trusted me." - Interviewee #20, Medical Director of a local clinic

Another important information-related code our team found was that of “misinformation and disinformation”. According to the American Psychological Association [7], misinformation is information that is false or inaccurate whereas disinformation, is false information that is created intentionally with the purpose to mislead through intentionally misstating factual true information. Out of the 19 interviews, our team had 43 counts for the code of misinformation and disinformation. Interviewees acknowledged that the issue of misinformation and disinformation in medicine existed prior to COVID. However, interviewees discussed how misinformation in general was exacerbated by the misunderstanding and misuse of professional research terminology by the public.

“I don't know just in general with misinformation, I mean it was already an issue in medicine before the pandemic and I think the pandemic just brought out all of our issues with misinformation because…we [scientists and researchers] use certain language with each other about things that I think doesn't always translate to the community” - Interviewee #2, Pediatric Endocrinologist

Other interviewees spoke of misinformation related to COVID vaccine risks that misconstrued the potential harms and the rise and spread of misinformation on social media.

“YouTube has been a horrible proponent of disinformation, a lot of them cite all of the YouTube conspiracy theorists that have really gotten major play during COVID.” Interviewee #4, Internal Medicine Doctor

The results of these different elements of information were the same: an increase of distrust in COVID vaccinations.

#### Secondary Data Analysis

The secondary data analysis focused on responses to the interview question, “What resources or tools would make these conversations easier for you?” as well as interview excerpts tagged with the codes “information gap” or “patient information needs.”

This yielded many ideas about what kinds of resources would help participants have better conversations with vaccine hesitant patients. Formats of interest ranged from handouts to providers reviewing materials with a patient, to short videos, websites, infographics and diagrams, advice on social media, testimonials from other patients, and waiting room literature, as shown by the quote below.

“common issues with the common concerns people have with the vaccines, like the best way to approach those might be helpful. Either as like in the form of a training or in the form of a handout that then we can summarize for patients” - Interviewee #2, Pediatric Endocrinologist

Participants also pointed out the value of curated information from authoritative and trusted local sources, such as the library or the health care system.

“it's good for the library to have something curated because there's a lot of information about that out there. And some form of curated information to again dispel misinformation about the effect of vaccines and people with long COVID” - Interviewee # 13, Integrative Medicine Doctor

“if UF had our own website I don't know if patients would trust that more versus the CDC because I feel like if it's political reasons, then, some people might feel like CDC is biased. So if it came through this institution where they get the majority of the healthcare, maybe it would be different.” - Interviewee #14, Internal Medicine Doctor

Participants indicated that resources should be catchy, printable, visual, colorful, eye-catching, and available in Spanish. For example,

“if, doctors and the CDC and all these people could have a little bit more pretty, catchy, things out there for why you should get the vaccine instead of boring websites” - Interviewee #2, Pediatric Endocrinologist

“I think generating more eye-catching infographics presenting simple facts in less technical language would be really effective.” Interviewee #9, Community Health Worker

They also addressed multiple constraints that were outside the scope of this project: time, additional training on communicating with patients, more vaccination locations in rural areas, and television commercials, among others.

#### Designing a Resource Guide

As a result of the secondary analysis, the team identified a need to gather key information in a single place. Given the lack of interview data about the specific mechanism for sharing resources, a LibGuide was chosen, as LibGuides are convenient, customizable, accessible, familiar to the project team, easily updated, and can lead to additional information in a way that paper resources cannot. While incorporating resources into the affiliated medical center’s electronic health record system would have been convenient for health care providers, the project team does not have direct access to the system, and this would have excluded community-based health and social service providers from accessing the resource.

The resources selected for review were all broad information portals from a variety of governmental and non-governmental sources. We anticipated that these sources would include a variety of types of resources designed for diverse audiences, so were most likely to meet our criteria. Resources reviewed included: the University of Florida HSCL and other libraries’ COVID-19 LibGuides [8], the Smithsonian’s Vaccines & US Campaign [9], the CDC-funded and Urban Institute-managed Partnering for Vaccine Equity Resource Hub [10], the University of Washington’s Center for Informed Public Resources page [11], the NNLM Misinformation Symposium [12], and the Office of the Surgeon General’s Community Toolkit for Addressing Health Misinformation [13].

In reviewing these clearinghouses and information portals, the team used the following criteria to identify specific resources for inclusion in the guide: a focus on COVID vaccines rather than general COVID information, primary sources of information rather than secondary, locally-developed sources created by trusted organizations where possible, sources addressing specific reasons for vaccine hesitancy identified in the interviews, designed for diverse audiences, and available in multiple languages.

Ultimately, this work resulted in the following resource guide: https://guides.uflib.ufl.edu/vaccinehesitancy.

In the interest of balancing a clean overall look and feel with including a useful selection of the best resources, the team designed the guide with two tabs, one for general vaccine hesitancy resources and one for resources specific to various reasons for vaccine hesitancy identified in the interviews (Figure 3). On the general resources tab, information is divided into two sections: one collection of resources for direct patient use and another of resources to help providers prepare before and after speaking with patients who may be hesitant to receive a COVID vaccine.

**Figure 3:**
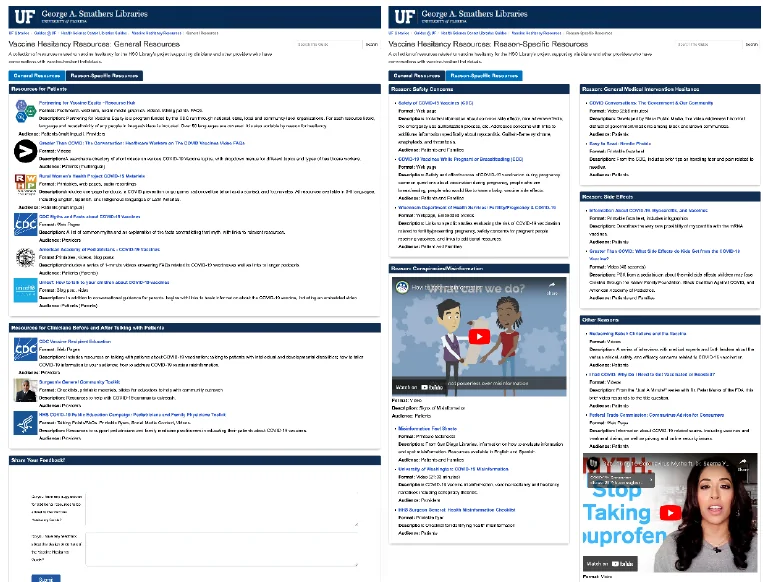
Screenshot of the vaccine hesitancy library guide pages.

The reason-specific resources tab identifies resources for each of the most frequent reasons for vaccine hesitancy that arose in the interview data. One of the team’s most significant challenges was finding authoritative resources that were accurate without being offensive or patronizing, especially ones addressing misinformation about the COVID vaccine.

Overall, the team limited the number of resources on each page to avoid overwhelming busy providers. Rather than being an inclusive page of every potential resource available, the goal was to have a more limited curated list of the most relevant and useful items. Similarly, only particularly high-quality embedded videos were included.

Local dissemination included email messages to primary care facilities and clinics, especially those working with underserved communities, and other organizations that provide vaccinations in a range of settings. This included groups like the Safety Net Collaborative, a group of more than 25 health organizations, primarily low and no-cost clinics, run by both University of Florida and community organizations. Dissemination also occurred through University of Florida patron groups and departments with which HSCL librarians liaise. These groups could use the guide themselves to provide information to their patients, as well as pass it along to students and other providers.

A goal of this project is continual improvement involving regular site maintenance with up to date and relevant information. Some of this maintenance will be addressed by the team with regular updates. The guide also contains an embedded feedback widget, giving users an opportunity to bring additional materials to public attention.

## DISCUSSION

### Content Analysis

The interviews provided rich data about many aspects of conversations between providers and their patients about vaccines. Of particular note were the themes of mis- or disinformation, trust, and information sources.

The quick and pervasive spread of mis- and disinformation about COVID and vaccines was described by many of the interviewed providers and is noted in the literature [14]. Sources of mis/disinformation included social media, friends, family, community members, and news outlets. This echoes findings in the literature related to the role of social media in exposing people to COVID-19 vaccine-related misinformation [15], as well the erosion of public trust in scientific recommendations during this time [16, 17]. The World Health Organization declared misinformation as a threat to public health in 2021 and this problem has become exacerbated since then by the rise of artificial intelligence [18]. The types of mis/disinformation mentioned during the interviews revolved around whether COVID existed, the potential treatments for COVID, and efficacy and safety concerns surrounding COVID vaccines. Interestingly, providers indicated a desire for a list of mis/disinformation types so they could be prepared when patients bring them up in conversation. This aligns with Bourgault’s recommended strategies for health providers to identify and navigate misinformation, some of which include “pre-bunking” and “debunking” misinformation using a combination of critical appraisal, trusted sources, and professional communication skills. [17] The importance of recognizing digital literacy and health literacy as significant challenges for patients has also been noted in the literature. To ameliorate this issue, multiple authors have developed or recommended frameworks to guide conversations about misinformation. [18, 19, 20, 21]

During the interviews, trust was discussed in the context of both the presence and absence of trust, most commonly in the context of the patient-provider relationship, but also in specific information sources. While the literature suggests that individuals’ level of trust towards their physicians consistently relates to their acceptance of vaccination [22], our interviewees had varying opinions about the amount of influence that providers have compared to relational and social sources of information. Some interviewees were confident in their authority as medical providers to influence patient decisions:

“She really just needed a doctor, somebody with a badge who could kind of speak in some plain language, just to explain to her what these vaccines really were. Because I could speak in an authoritative way, I seemed like I really did know what I was talking about; she felt she could trust me more than the YouTube video that she saw and that was enough." - Interviewee #20, Medical Director of a local clinic

The potential influence of healthcare providers as trusted authorities is echoed in the literature: Wynn encourages nurses to engage patients in the online spaces where they may be getting health information, in order to better understand patients’ information environment and promote trustworthy sources of information. [21]

Other interviewees felt that their advice was being disregarded in favor of other relational and social sources:

“Helping to educate family members who have that influence on their college age kids I think would be helpful…because I don't know if there would really be any source that they would believe over something their parents said or their friends said or something they read on Twitter or Facebook." - Interviewee #5, Doctor at a university student health care center

In contrast to this interviewee’s apprehension, both Sharman and Southwell encourage providers to elicit information from their patients about potential other sources of information about conditions and treatments, as an opportunity to learn more about the patient’s perspective and to start a conversation where misinformation can be addressed. [23, 24]

Providers also described struggles with sharing health information from traditional authoritative sources because of the distrust that patients had developed, particularly of governmental sources, over the course of the pandemic.

For example,

“I've given them the FDA fact sheets on the vaccines I've given them CDC COVID 19 resources, but they don't trust those resources. If they're vaccine hesitant, whatever resources I may give them they don’t know that they trust them because they're not sure if they came from the FDA or the CDC- they've already written those agencies off." - Interviewee #4, Internal Medicine Doctor

This was not limited solely to the United States; Abbasi et al found in their interviews with British providers a perception that patients felt the government was telling them what to do to the extent they felt they had no voice or agency, eroding trust in public health officials even more. [25]

### Limitations

The team sought a diverse sample of participants to interview, as the project scope purposefully defined “provider” very broadly, but ultimately half of the participants were faculty from the University of Florida College of Medicine. The limitations from our participant demographics have skewed our findings towards mainly academic providers in the College of Medicine. We know that vaccine hesitancy is an issue that requires interprofessional collaboration and although our team tried to solicit participants from other profession to provide a much-needed holistic view, we were unable to. As is typical of many library-based research projects, the sample also consisted of those with pre-existing relationships to the library which creates another limitation; the opinions of providers who do not make use of library resources could differ.

## CONCLUSION

This project provided valuable lessons on best practices for efficiently conducting team qualitative research (data collection and analysis), yielded interesting insights regarding patient-provider communication, and informed the creation of a useful provider-focused resource guide.

This project also showed the importance of health sciences librarians conducting and participating in research to support the development of patient education information resources for providers, especially in response to rising public health concerns. Vaccine hesitancy, combined with rapid misinformation, is a growing threat to public health and wellbeing [26]. Providers and patients should collaborate with information professionals to find vetted, verified, and quality resources to help the public make informed unbiased decisions about their health.

In the future, the team will share a detailed description of the methodology used in this project for the use of other librarians and qualitative researchers. Other team goals are to continue disseminating the resource page and to conduct usability testing to maximize value.

## Data Availability

The interview protocol, coding protocol, and coded, anonymized transcripts are available through the project’s repository on Open Science Framework: https://osf.io/f875j/?view_only=7d01a78c7a15481c80e9e94229ac329f. Some of the quotes in the manuscript received minor editing to remove repetition.

## SUPPLEMENTAL FILES

**Appendix A:** Interview Session Protocol
