# Supplementary material for: Evidence-based design of vaccine hesitancy information resources for health care providers
Source: J Med Libr Assoc. 2026 Oct 14;114(4):452–61. doi: 10.5195/jmla.2026.2135 (PMC13670223; doi:10.5195/jmla.2026.2135)
Supplement: Supplementary file 1 [file jmla-114-4-452-s01.pdf]

Protocol For Vaccine Hesitancy Interviews/Focus Groups

Prior to Session

1. After potential participants respond to survey with their preferred times, identify best time slot and contact the designated librarian/student available for that time slot.
2. Student Research Assistant (or librarian, if time slot is one with two librarians facilitating) schedules Zoom session.
    a. Be sure to adjust recording settings in Zoom prior to the session (you want to check/uncheck so your settings to look like the ones below):

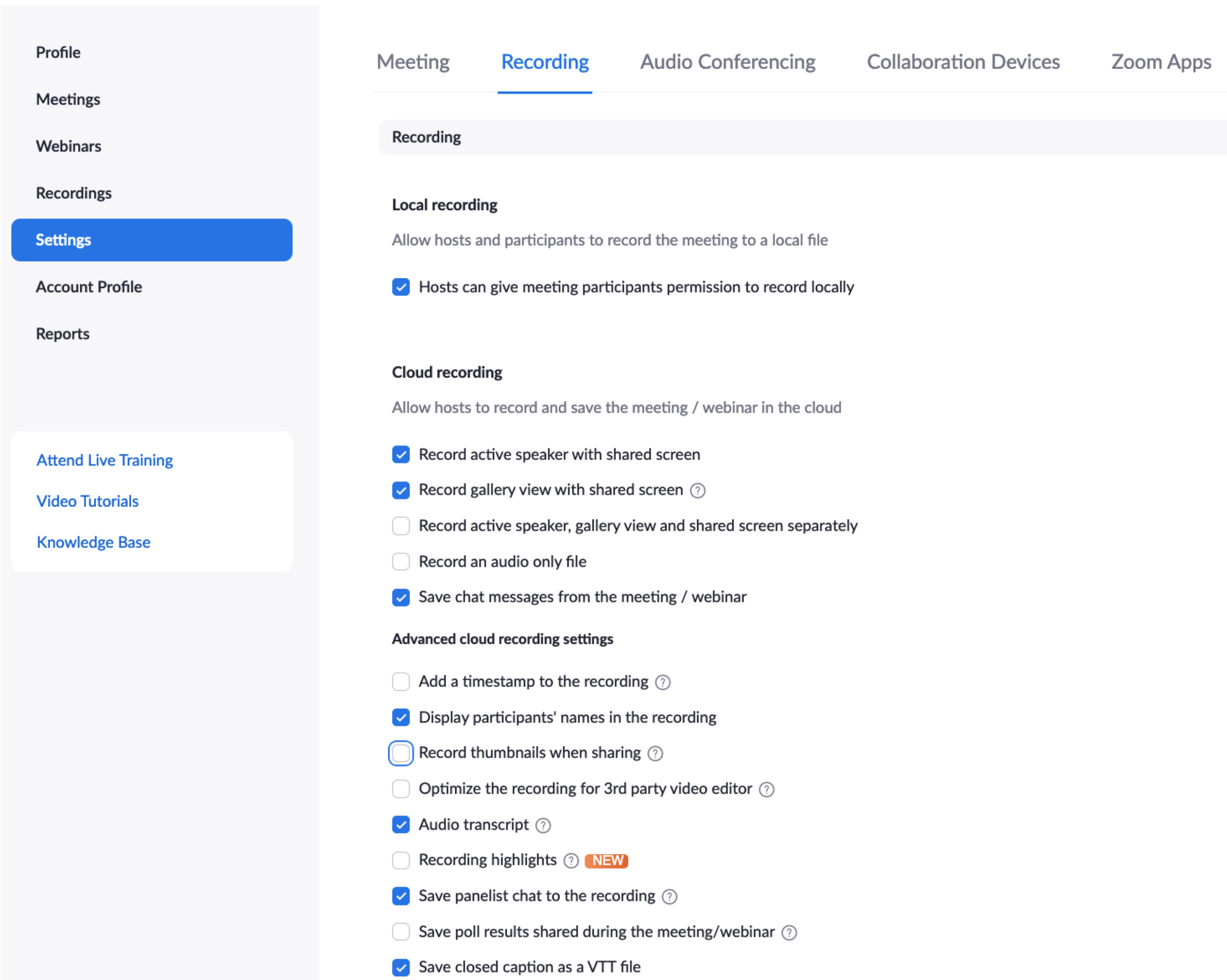

3. Interview Coordinator emails participant a confirmation with the date and time of the session, a PDF copy of consent form for review, and the Zoom link (copying the [other] librarian).
4. Interview Coordinator emails the participant a reminder the day before the scheduled interview.

Session

ARRIVE 10 MINUTES EARLY INTO THE ZOOM ROOM

Introduction:

"Hi [name], thanks for joining."

"I am [XXX]."

"Before we begin, is [name] the correct way to pronounce your name?"

"OK, thanks."

"We will be recording your voice & image for the session. I also have a colleague [introduce note taker here] here with me. They'll just be quietly observing the session and taking notes."

"Does that all sound ok with you?"

Confirming Informed Consent:

[Share the consent form: https://ufl.qualtrics.com/jfe/form/SV_6VZFGgtA536iqgu]

This is the consent form that was provided when you signed up to participate in the study. It explains what we're doing here today and how we will use the information. Please take a moment to review.

[pause]

Do you have any questions?

If you still agree to participate and have no further questions, please place your name in the box and select the option to Agree and then click submit. We will then proceed with the session.

[Confirm affirmative response in *Qualtrics* before proceeding.]

Recording:

"Is it ok if we begin recording?"

[Wait for confirmation, then begin recording **to the cloud**.]

Session Instructions:

"Ok great. So the purpose of this interview is to learn more about your experiences talking about vaccines with patients/community member that you interact with in a professional capacity. We have a few questions that we'll be asking related to this topic. We'd like you to focus on COVID-19 vaccine conversations, particularly conversations in which the patient/community member expressed hesitance about the vaccine, but any stories or information you can share about how you navigate vaccine hesitancy in general will be useful. At no time will we ask you the identity of any of your patients. You are also welcome to decline answering any of the questions we ask, or to leave the interview at any time."

Participant Introduction:

"Could you introduce yourself? What is your current job/profession? Where do you work?"

Common Questions (that we'll ask at each session)

[For each question, copy and paste the question into the chat, and then read it aloud.]

Core Questions:

1. Do you talk to your patients about getting vaccines for: 1)Themselves 2)Their children, or 3)Other loved ones (parents, etc)? How do you approach these conversations?
2. Do you encounter people who hold beliefs that may make them hesitant to get vaccines? What strategies do you use to discuss their beliefs with them?
3. Where do you think patients receive the most influential information about vaccines from?
4. What factors have you seen/ do you think influence a person's decision on whether to get a vaccine or not?
5. How do you decide which resources are most suitable to recommend for particular patients, in relation to vaccines?
6. In your opinion, what are the best ways to present vaccine information to patients?
7. What resources or tools would make these conversations easier for you?

Optional Questions:

- How could awareness of the importance of vaccinations be spread?
- How do you engage patients in the vaccine decision-making process?
- Do you encounter vaccine hesitancy in people you work with in your role?
- Do you have difficult conversations with people about their health or the health of their families, friends, students, etc?
- Do you take different approaches depending on their reason for hesitancy?
- What approach do you take if you want them to understand certain facts or to change their minds?
- What kinds of information sources do you suggest to vaccine hesitant patients, if any?
- Do you want to "change their minds" or enable them to have their say and explain their beliefs?
    - If they decline to get a vaccine, do you ask them why?
    - What are reasons given for their vaccine hesitancy?
    - Do these reasons differ depending on the type of shot? Who is getting it?

Stop Recording

[Stop recording]

Compensation

"Thank you for taking the time to participate in the study"

"I just need your UFID to issue your gift card. It will be sent to your email in the next couple of weeks."

*If the person is not from UF, please ask for Legal Name, Mailing Address, Email Address, and Contact Phone Number.

After the Session Tasks

Debrief immediately following session if necessary.

Next steps:

1. Edit transcript in Zoom to correct any typos so that the transcript accurately reflects the conversation.
2. Downloads the transcript from Zoom and put in the HSCL shared drive.
